# Aortic Prosthesis-Associated MDR *Pseudomonas* Infections as a Diagnostic and Therapeutic Challenge

**DOI:** 10.3390/idr12030012

**Published:** 2020-11-02

**Authors:** Rita Filipe, Filipa Ceia, Ana Cláudia Carvalho, Margarida Tavares, José Teixeira, António Sarmento

**Affiliations:** 1 Infectious Diseases Department, São João University Hospital Center, 4000-319 Porto, Portugal; filipa.ceia@chsj.min-saude.pt (F.C.); aclaudia.carvalho@chsj.min-saude.pt (A.C.C.); margarida.tavares@chsj.min-saude.pt (M.T.); antonio.sarmento@chsj.min-saude.pt (A.S.); 2 Faculty of Medicine, University of Porto, 4000-319 Porto, Portugal; 3 EPI Unit, Public Health Institute of Porto University, 4000-319 Porto, Portugal; 4 Vascular Surgery Department, São João University Hospital Center, 4000-319 Porto, Portugal; jteixeira@chsj.min-saude.pt

**Keywords:** aortic prosthesis infection, MDR *Pseudomonas*, PET scan, axilobifemoral revascularization

## Abstract

Endovascular prostheses are used to treat life-threatening conditions such as ruptured aortic aneurysms. Prosthetic infection cause significant morbidity and mortality, posing important diagnostic and therapeutic challenges. It is particularly difficult to diagnose and, in the era of multidrug resistance (MDR), these type of infections may become even more difficult to treat. Herein, we reported a case of a secondary prosthetic endovascular infection following repeated bacteremia episodes from a urinary source. This case illustrates an MDR *Pseudomonas aeruginosa* aortic infection that was difficult to diagnose with no oral antibiotic treatment options.

## 1. Case Report

In 2015, a 59-year-old man underwent emergency surgery to repair a ruptured abdominal aortic aneurysm. An aortobifemoral bypass was performed. As a post-operatory complication, the patient developed a left retroperitoneal hematoma and secondary hydronephrosis, which was managed with a J-J catheter placement. In the following year, he had several episodes of catheter-associated pyelonephritis with repeated isolation of multidrug resistance (MDR), *Pseudomonas aeruginosa* in urine, and, on one occasion, blood cultures. *P. aeruginosa* was resistant to ciprofloxacin (minimum inhibitory concentration (MIC) 4 µg/mL), gentamicin (MIC 8 µg/mL), and imipenem (MIC 16 µg/mL); sensitive to amikacin, ceftazidime, tobramycin, cefepime, and colistin; and it showed intermediate resistance to aztreonam. MIC were extrapolated data from VITEK^®^2.

On December 2016, he was admitted in our Department of Infectious Diseases, which has 27 beds and is located at São João Hospital, a tertiary care public university hospital in Oporto, Portugal, with 1100 beds and about 45,500 admissions per year. At that time, he was admitted with another episode of pyelonephritis and J-J catheters were removed. The patient was treated with IV targeted therapy for 14 days (amikacin 1 g daily and ceftazidime 2 g every 8 h) with clinical and analytical improvement. 

Following repeated episodes of infections with the same microorganism, we suspected an infection of the aortobifemoral bypass. An echocardiogram was normal and a full body LeukoScan^®^ did not detect radiolabeled antibodies. Follow-up blood cultures were also negative. 

**Figure 1:**
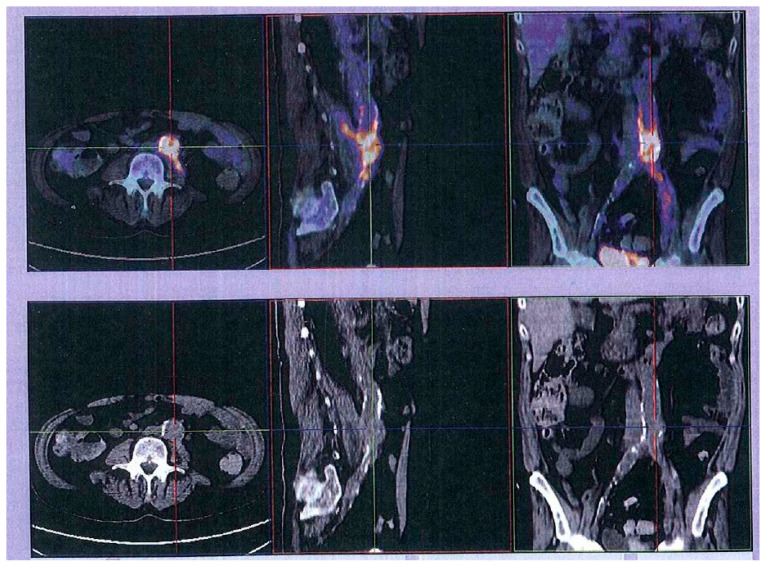
18F-FDG PET/CT revealing hypercaptation along aortic prosthesis extending into common and left external iliac arteries.

Four days after discharge, the patient was readmitted with fever and peripheral embolization in the inferior limbs. Amikacin and ceftazidime were started. MDR *P. aeruginosa* was again isolated in urine and blood cultures. *Pseudomonas* was resistant to ciprofloxacin (MIC 4 µg/mL), gentamicin (MIC 8 µg/mL), imipenem (MIC 16 µg/mL), and piperacillin-tazobactam (MIC 128 µg/mL); sensitive to amikacin, ceftazidime, cefepime, tobramycin, and colistin; and with intermediate resistance to aztreonam. Angio-computerized tomography (CT) showed that there was a fluid collection adjacent to the left psoas muscle with contrast uptake in the collection walls, apparently in continuity of the bypass bifurcation region. This had previously been detected in a post-operatory exam with similar dimensions, suggesting postoperative hematoma. However, given the high suspicion of bypass infection, a LeukoScan^®^ was repeated but again showed no changes. A PET scan was then requested and showed increased 18F-fluorodeoxyglucose (FDG) along the aortic prosthesis to the common iliac artery and external iliac artery, with superior intensity at the distal portion of the aorta and extending to the left psoas muscle suggesting an infectious process (Figure 1). 

Suppressive antibiotic treatment was considered given the high morbidity and mortality associated to prosthesis removal but due to *Pseudomonas*, there were no possible oral antibiotics. After consulting the vascular surgeon, the patient underwent aorta-bifemoral bypass removal and extra anatomic revascularization, with a silver impregnated axilobifemoral graft. Aortic graft was placed in thioglycolate broth for 48 h which was then transferred into a blood agar plate. Culture from the graft confirmed the infection with an isolate of *P. aeruginosa*, with the same phenotypical profile. 

The patient was discharged after completing 10 weeks of antibiotic therapy and remained free of infection for the next 18 months of follow-up. 

Of note, in October 2018, he developed a catheter-associated urinary tract infection caused *P. aeruginosa* with the same antimicrobial susceptibility profile, treated successfully with ceftazidime for two weeks. There were no infectious complications in the following year. The patient was also admitted into the Vascular Surgery Department for three episodes of bypass occlusion for the last two years that were treated with thrombolytic agents. 

## 2. Discussion

Aortic graft infections (AGIs) are defined as infections of any vascular and endovascular grafts implanted from subdiaphragmatic aorta to the groin [1]. 

The reported rate of AGI is below 1% in most series, and it is significantly associated with hematogenous spread and surgical site infection [2,3]. However, the incidence is higher when considering only patients treated in the emergency setting and when there is an open surgery [1,2]. In fact, in some reports, AGIs range from 0.6–3% [1].

There are multiple recognized risk factors for endograft infections: prolonged operative time, perioperative antiseptic technique defects, nasal carriage of *Staphylococcus aureus*, perioperative infection in another site, older age, and chronic diseases. The most important cause of endograft infections is surgical site infection [1]. 

Besides typical symptoms of systemic infections, diagnosis relies on microbiological diagnosis and imaging findings. However, up to a third of patients with endograft aortic prosthetic infections may present negative blood cultures or negative cultures of the graft/drainage material, thus not having a microbiological diagnosis [2]. When microbiological identification is possible, coagulase-negative Staphylococcus is the most common agent, followed by *Pseudomonas aeruginosa*, Enterococcus spp. and Staphylococcus aureus [4]. This reflects the most common sources of contamination: skin and adjacent bowel. 

Multi-drug resistant (MDR) organisms are an increasing concern and when heterologous material is involved, biofilm may hinder blood sterilization and impair clinical response. It is known that biofilm formation, a complex bacteria network protected in a matrix, can develop in device associated infections and contribute to the survival of bacteria. Bacterial adherence depends on several factors such as the length of stent graft in contact with the arterial wall and on the extent of endothelialization of the stent graft surface by mature collagen tissue [5]. 

Angio-CT is considered the gold standard for endograft infections because of its high sensitivity (approaching 100% in most cases, but which decreases to 55% in late or low-grade infections) and specificity (100%) [1,5]. Positron emission tomography (PET)-CT and leukocyte scan may be useful to establish the diagnosis [5]. Of note, LeukoScan^®^ is often falsely negative in long standing infections and in patients with repeated courses of antibiotics, as in our case. In our patient, PET was decisive to diagnosis. A study that included 76 patients and a total of 96 prosthetic grafts concluded that PET-CT gave reliable results with an accuracy >95% [6].

Because of its low incidence, management of aortic endograft infections is not straightforward and should be evaluated individually. Possible options include endovascular aortic repair, which may impel graft removal, open surgery, or conservative treatment. The latter consists of long term oral suppressive treatment and this is usually considered in high risk patients unfit for surgery, in which, ideally, antibiotic therapy should be directed to an isolated microorganism [7]. Additionally, when there are no oral antimicrobial options, nor parenteral regimens suitable for a parenteral outpatient administration, lifelong treatment may not be practicable. The duration of antibiotic treatment is not widely agreed with. 

Mortality associated with endograft infections is variable and ranges from 30–60%, being typically higher in patients treated conservatively [1]. Thus, consensus is that infected material should always be removed, if feasible. Surgical treatment consists of graft removal and revascularization.

In the particular case of *Pseudomonas* graft infection, many authors believe that graft preservation is not an option as outcomes are even worse than for other agents [8]. In a study that included 9 cases of *Pseudomonas aeruginosa* infections, successful graft preservation was accomplished in only 44% (4 of 9) of cases, compared to 70% and 92% of cases when Gram positive bacteria or other Gram-negative bacteria were cultured, respectively [4]. 

However, in-hospital mortality in patients that undergo graft excision is significant, reported to be as high as 48% [9], and in some cases technically very difficult. In our patient, this was an obstacle for a quicker decision for open surgery. Our patient survived complex surgery and according to the literature survival rate ranges from 63–73% at 3 years of follow-up [10,11]. 

## 3. Conclusions

Our case highlights the importance of maintaining a high suspicion of endovascular infection if a patient with a vascular graft has persistent bacteremia even if the imaging tests do not contribute to its confirmation. In our patient, Angio-CT and LeukoScan^®^ had limited value and it was the PET scan which was decisive. Persistence and clinical judgment are essential when pursuing endovascular infection diagnosis. 

On the other hand, the decision to remove an aortic graft, a surgery that implies a high mortality, may be difficult but may be the only curative strategy. Although the isolation of a microorganism allowed direct antibiotic therapy in this case, bacteremia persisted while graft was in place. 

Due to the challenges that these infections pose, the management of these patients should be carried out in multidisciplinary teams that include vascular surgeons and infectious disease specialists.
